# Luteolin Confers Cerebroprotection after Subarachnoid Hemorrhage by Suppression of NLPR3 Inflammasome Activation through Nrf2-Dependent Pathway

**DOI:** 10.1155/2021/5838101

**Published:** 2021-11-05

**Authors:** Zi-Huan Zhang, Jia-Qiang Liu, Cheng-Di Hu, Xin-Tong Zhao, Fei-Yun Qin, Zong Zhuang, Xiang-Sheng Zhang

**Affiliations:** ^1^Department of Neurosurgery, The First Affiliated Hospital of Wannan Medical College (Yijishan Hospital of Wannan Medical College), Wuhu 241001, China; ^2^Department of Neurosurgery, Wuwei People's Hospital, Wuwei City, 238399, China; ^3^Department of Neurosurgery, Nanjing Drum Tower Hospital, The Affiliated Hospital of Nanjing University Medical School, Nanjing, 210008 Jiangsu, China; ^4^Department of Neurosurgery, Beijing Friendship Hospital, Capital Medical University, Beijing 100050, China

## Abstract

Luteolin (LUT) possesses multiple biologic functions and has beneficial effects for cardiovascular and cerebral vascular diseases. Here, we investigated the protective effects of LUT against subarachnoid hemorrhage (SAH) and the involvement of underlying molecular mechanisms. In a rat model of SAH, LUT significantly inhibited SAH-induced neuroinflammation as evidenced by reduced microglia activation, decreased neutrophil infiltration, and suppressed proinflammatory cytokine release. In addition, LUT markedly ameliorated SAH-induced oxidative damage and restored the endogenous antioxidant systems. Concomitant with the suppressed oxidative stress and neuroinflammation, LUT significantly improved neurologic function and reduced neuronal cell death after SAH. Mechanistically, LUT treatment significantly enhanced the expression of nuclear factor-erythroid 2-related factor 2 (Nrf2), while it downregulated nod-like receptor pyrin domain-containing 3 (NLRP3) inflammasome activation. Inhibition of Nrf2 by ML385 dramatically abrogated LUT-induced Nrf2 activation and NLRP3 suppression and reversed the beneficial effects of LUT against SAH. In neurons and microglia coculture system, LUT also mitigated oxidative stress, inflammatory response, and neuronal degeneration. These beneficial effects were associated with activation of the Nrf2 and inhibitory effects on NLRP3 inflammasome and were reversed by ML385 treatment. Taken together, this present study reveals that LUT confers protection against SAH by inhibiting NLRP3 inflammasome signaling pathway, which may be modulated by Nrf2 activation.

## 1. Introduction

Subarachnoid hemorrhage (SAH), a detrimental type of stroke, is considered a life-threatening disease with limited therapeutic options [1]. There are urgent unmet needs for intervention that could block progressive brain damage after SAH. Recently, numerous clinical and experimental studies have suggested that the robust reactive oxygen species (ROS) overproduction and neuroinflammation play important roles in the secondary brain injury after SAH and contribute greatly to the neurological deficits [2–5]. Accordingly, identifying new and effective therapeutic strategies to mitigate excessive oxidative damage and neuroinflammation is a pressing need.

Luteolin (LUT) is an abundant flavonoid distributed in vegetables and fruits such as broccoli and carrots [6]. Previous reports have demonstrated that LUT is a neuroprotective flavonoid by scavenging free-radical and inhibiting inflammation in a series of neurological disorders, including traumatic brain injury (TBI), ischemic stroke, and Alzheimer's disease [6–8]. Moreover, LUT can easily penetrate the blood-brain barrier and improve behavioral performance in acute brain injuries [8, 9]. However, whether LUT could exert cerebroprotective effects against SAH remains unknown.

There is emerging evidence indicates that nod-like receptor pyrin domain-containing 3 (NLRP3) inflammasome-mediated neuroinflammation plays a critical role in the development of secondary brain injury after SAH [10–13]. In addition, ROS generation is closely associated with oxidative damage and is one of the major signals that trigger the NLRP3 inflammasome activation. Nuclear factor-erythroid 2-related factor 2 (Nrf2) is considered as the most important endogenous factor in the maintenance of cellular homeostasis. Under the condition of oxidative stress, Nrf2 translocates into the nucleus and binds with an antioxidant response element (ARE), thereby promoting a battery of antioxidative gene expressions, such as heme oxygenase-1 (HO-1), glutathione peroxidase (GSH-Px), and superoxide dismutase (SOD) [14–16]. Accumulating studies have indicated that activating the Nrf2 signaling pathway plays a key role in attenuation of oxidative damage in a variety of oxidative disorders [8, 17]. Moreover, recent studies have suggested that NLRP3 inflammasome activation is modulated by Nrf2 signaling [18, 19]. Intriguingly, LUT is a powerful Nrf2 activator and can inhibit NLRP3 inflammasome in a series of disease models [8, 20, 21]. Thus, in this study, we investigated whether LUT had therapeutic potential in SAH and verified whether the beneficial effects of LUT were associated with the inhibition of NLRP3 inflammasome activation by Nrf2-dependent pathway.

## 2. Materials and Methods

### 2.1. Animals

All of the procedures were approved by the Institutional Animal Care and Use Committee of Wannan Medical University and met the accordance of National Institutes of Health. Health adult male Sprague Dawley rats (250–300 g) were bought from the Nanjing Biomedical Research Institute of Nanjing University. The animals had free access to food pellets and water ad libitum.

### 2.2. SAH Model

A prechiasmatic cistern injection model was used in our study. Anesthesia was induced by intraperitoneal injection avertin (200 mg/kg). After anesthetization, rats were positioned prone in a stereotactic frame. And then, a burr hole was drilled into the skull 7.5 mm anterior to the bregma. A total of 0.35 mL of nonheparinized fresh autologous arterial blood was retracted from the femoral artery and injected into the burr hole (in the course of 20 s) under aseptic conditions [22]. Bone wax was employed to block cerebrospinal fluid leakage. Sham operation animals were injected with 0.35 mL physiological saline instead of blood into prechiasmatic cistern. Consistent with previous studies [17, 23], the inferior basal temporal lobe always contained blood and differed histologically from control rat brain. Therefore, the basal temporal lobe adjacent to the clotted blood was used for histopathologic examination in the current study.

### 2.3. Study Design

In the first set of experiments, 108 rats (127 rats were used, and 19 rats died) were divided into the following groups: sham (*n* = 14), sham+vehicle (*n* = 16), SAH+vehicle (*n* = 20, 5 rats died), SAH+10 mg/kg LUT (*n* = 20, 5 rats died), SAH+30 mg/kg LUT (*n* = 19, 3 rats died), SAH+60 mg/kg LUT (*n* = 19, 3 rats died), and SAH+90 mg/kg LUT (*n* = 19, 3 rats died) groups. Rats were killed at 24 h and 72 h after operation. Post-assessments included behavior performance, brain water content, and histopathological study.

In the second set of experiments, 48 rats (57 rats were used, and 9 rats died) were assigned into four groups: sham+vehicle (*n* = 12), SAH+vehicle (*n* = 15, 3 rats died), SAH+60 mg/kg LUT (*n* = 14, 2 rats died), and SAH+60 mg/kg LUT+ML385 (*n* = 16, 4 rats died) groups. Rats were killed at 24 h after SAH. Post-assessments included immunofluorescence staining, biological estimation, and behavior performance.

### 2.4. Primary Cell Culture and In Vitro SAH Model

Primary cortical neurons and microglia were performed according to our previous study [24]. Rat pups were sacrificed on postnatal days 0-1. For primary neurons culture, cortical cells were cultured onto poly-D-lysine-coated plates and suspended in neurobasal media supplemented with B27, glutamate, Hepes, penicillin, and streptomycin. For primary microglia culture, cortical cells were placed in serum-free DMEM-F12 culture medium. Regarding the neurons and microglia coculture system, microglia were seeded in transwell upper chamber (Corning, pore size = 0.4 *μ*m) and the neurons were cultured in the plates. Coculture medium was DMEM with 10% FBS.

To mimic SAH in vitro, the coculture system was stimulated with 10 *μ*M oxyhemoglobin (OxyHb) for 24 h. The dose of OxyHb was chosen according to our previous study [23]. Hemoglobin (Sigma, USA) was used to produce OxyHb as we described in detail before [25]. The neuron–microglia cocultures were partitioned into the following groups: control, oxyHb, OxyHb+5 *μ*M LUT, OxyHb+10 *μ*M LUT, OxyHb+25 *μ*M LUT, and OxyHb+25 *μ*M LUT+ML385. The coculture system was harvested 24 h after indicated intervention. The culture medium, neurons, and microglia were collected for cell viability analysis, biochemical estimation, and immunofluorescence staining.

### 2.5. Drug Administration

For in vivo experiments, LUT (Sigma-Aldrich, St. Louis, MO, USA) was dissolved in dimethylsulfoxide (DMSO) (<1%) and physiological saline at different concentrations (10, 30, 60, and 90 mg/kg). Rats were intraperitoneally administrated with LUT at different doses at 2 h after insults and then once daily until euthanasia. ML385, a selective Nrf2 inhibitor, was dissolved in DMSO and physiological saline and was administered intraperitoneally (30 mg/kg) for 2 days before SAH induction. For in vitro experiments, LUT (5 *μ*M, 10 *μ*M, and 25 *μ*M) and ML385 (10 *μ*M) were dissolved in 0.1% DMSO (in physiologic saline) and then added to culture medium [8, 26]. LUT was administered for 0.5 h prior to OxyHb incubation. Cells were pretreated with ML385 24 h before OxyHb stimulation.

### 2.6. Neurological Behavior

Neurologic functions were recorded using an 18-point scoring system reported by Zhao et al. [21]. Rotarod test was used to assess motor function according to a previous study [16]. The rotating speed was gradually increased from 4 to 40 rpm over a 5 min period. The latency to fall was recorded. The mean latency was calculated based on three consecutive trials.

### 2.7. Brain Water Content

In brief, the brains were harvested immediately after sacrificing the rats and dissected into cerebrum, cerebellum, and brainstem. Each part was weighed to record the wet weight. The dry weight was recorded after the samples were being dried at 80°C for 72 h. Brain edema ratio was calculated as [(wet weight–dry weight)/wet weight] × 100%.

### 2.8. Nissl Staining

In brief, the brain tissue was first fixed in 4% paraformaldehyde for 48 h and then performed as we described in detail before [17]. Afterward, tissue sections were stained with Nissl solution for 10 min and mounted with Permount. All the sections were photographed in the microscope.

### 2.9. Immunofluorescence Staining

Immunofluorescence staining was performed as we described in detail before [17]. In brief, the sections were first fixed by 0.1% Triton X-100 and then were blocked by 5% BSA for 2 h. After three washes with PBS, brain tissues or cultured neurons were incubated with primary antibodies against Iba-1 (1: 50, Santa Cruz Biotechnology or 1 : 100, Abcam), myeloperoxidase (MPO, 1 : 50, Santa Cruz Biotechnology), 8-hydroxydeoxyguanosine (8-OHdG) (1 : 100, Abcam), Nrf2 (1 : 100, Abcam), caspase-1 p20 (1 : 50, Santa Cruz Biotechnology), and NeuN (1 : 200, EMD Millipore). Sections were then incubated with corresponding secondary antibodies (Alexa Fluor 488 and Alexa Fluor 594) overnight at 4°C. Fluorescence microscopy imaging was examined under a ZEISS HB050 inverted microscope system. The fluorescently stained cells were recorded using Image J program.

### 2.10. TUNEL Staining

TUNEL staining was detected by a terminal deoxynucleotidyl transferase-mediated dUTP nick-end labeling (TUNEL) detection kit (Roche Inc., Indianapolis, USA) in line with the manufacturer's instructions. The brain slices and coverslips were incubated with the primary antibody anti-NeuN (1 : 200, EMD Millipore) overnight. And then, a reaction solution and converter-AP were incubated subsequently. The fluorescently stained cells were recorded using Image J program.

### 2.11. ELISA

Brain samples and culture medium were collected. The levels of IL-1*β*, tumor necrosis factor-*α* (TNF-*α*), and IL-6 were evaluated in line with the manufacturer instructions (Multi Sciences. China).

### 2.12. Biochemical Estimation

The contents of malondialdehyde (MDA), GSH-Px, glutathione (GSH), and SOD were evaluated by using commercially available kits in line with the manufacturer instructions (Nanjing Jiancheng Bioengineering Institute, Nanjing, China). For MDA, brain samples were mixed with thiobarbituric acid, acetic acid, and sodium dodecyl sulphate at 95°C for 30 min. And then, the sample was centrifuged at a rate of 4,000 rpm for 10 min. The MDA contents in all tubes were measured at the wavelength of 532 nm using a spectrophotometer. For H_2_O_2_, the fresh brain tissue was homogenized using the assay buffer and processed per kit instructions (Abcam). Absorbance at 590 nm was recorded. For GSH-Px, the supernatant was mixed with sodium azide, glutathione reductase enzyme, NADPH, and H_2_O_2_. The GSH-Px activity was measured at the wavelength of 340 nm using a spectrophotometer. For GSH, the supernatant was mixed with 1% trichloroacetic acid and centrifuged at 10,000 × g for 15 min. The reaction mixture consisted of lysates and 5,5′-dithiobis-(2-nitrobenzoic acid). GSH was measured at the wavelength of 405 nm. For SOD, sample was mixed with the xanthine and xanthine oxidase in potassium phosphate buffer at 37°C for 20 min. SOD was measured at the wavelength of 450 nm using a spectrophotometer.

### 2.13. Western Blotting

The brain samples and the primary microglia in transwell upper chambers and neurons in the plates were collected for Western blotting. In brief, the extracted proteins were separated by Tris-glycine SDSPAGE and then transferred to PVDF membranes for 30 min. Primary antibodies used were Nrf2 (1 : 1000, cat# ab31163, Abcam), HO-1 (1 : 1000, cat# ab13243, Abcam), NLRP3 (1 : 200, cat# SC-66846, Santa Cruz Biotechnology), adaptor apoptosis-related speck-like protein (ASC) (1 : 200, cat# SC-22514, Santa Cruz Biotechnology), caspase-1 (1 : 200, cat# SC-56036, Santa Cruz Biotechnology), caspase-1 p20 (1 : 200, cat# SC-398715, Santa Cruz Biotechnology), Histone H3 (1 : 3000, cat# BS7416, Bioworld Technology), and *β*-actin (1 : 3000, cat# AP0060, Bioworld Technology, Minneapolis, MN, USA). Then, the membranes were incubated for 2 h at room temperature with corresponding second antibody. Detection was conducted by using chemiluminescence solution.

### 2.14. Cell Viability Analysis

Cell viability was detected by the cell counting kit- (CCK-) 8 assays or lactate dehydrogenase (LDH) activity with commercially available kits (Beyotime Biotechnology, China) in accordance with the manufacturer's instructions.

### 2.15. DCFH-DA Staining and Propidium Iodide Staining

For ROS measurements, primary cultured neurons were incubated with 2,7-dichlorodihydrofluorescein diacetate (DCFH-DA, Sigma) for 10 min at 37°C. For propidium iodide (PI) staining, the primary neurons were stained with 2 *μ*g/mL of PI for 10 min at 37°C. Quantifications were performed with Image Pro Plus 6.0.

### 2.16. Statistical Analysis

Statistical software GraphPad Prism 8.02 (GraphPad Software, La Jolla, CA, USA) was used for statistical analysis. All data were expressed as mean ± SD. Differences among multiple groups were compared by one-way or two-way analysis of variance with Bonferroni post hoc test. Statistical significance was inferred at *P* <0.05.

## 3. Results

### 3.1. Dose-Response Effects of LUT on SAH

**Figure 1:**
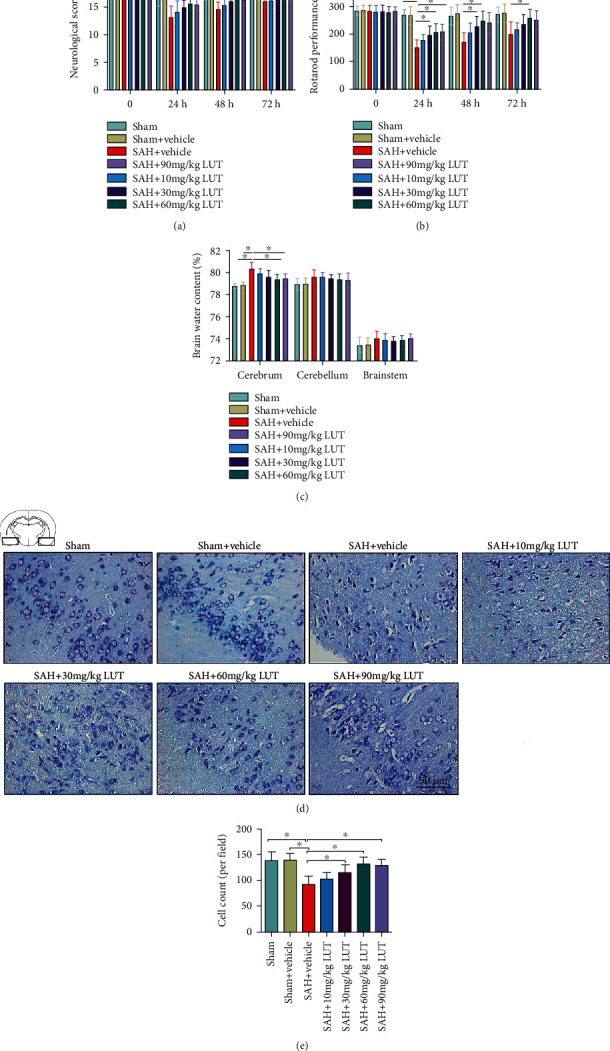
Dose-response effects of luteolin (LUT) on subarachnoid hemorrhage (SAH) in rats. Effects of three LUT doses on (a) neurologic scores (*n* = 8‐10 per group), (b) rotarod performance (*n* = 8‐10 per group), and (c) brain water content (*n* = 6 per group). (d) Representative photomicrographs of Nissl staining at 72 h after SAH. (d) Magnification ×200, scale bar 50 *μ*m. For Nissl staining, the basal temporal lobe adjacent to the clotted blood was evaluated. (e) Quantification of the proportion of surviving neurons at 72 h after SAH (*n* = 6 per group). Bars represent the mean ± SD. ^∗^*P* < 0.05.

No animals died in the sham and sham+vehicle groups. The mortality rate of the rats was 22.9% (8 of 35) in the SAH+vehicle group, 17.6% (16 of 91) in the SAH+LUT group, and 25% (4 of 16) in the SAH+LUT+ML385 group. In a dose-response study, LUT was administered to rats after SAH at 10, 30, 60, and 90 mg/kg. Doses of 30 mg/kg, 60 mg/kg, and 90 m/kg, but not 10 mg/kg, markedly improved neurologic scores and rotarod performance in the early period after SAH (Figures 1(a) and 1(b)). In addition, LUT treatment at 60 mg/kg and 90 m/kg, but not 10 mg/kg and 30 mg/kg, significantly reduced brain water content in cerebrum after SAH (Figure 1(c)). Nissl staining further showed that evident damage was seen in the SAH+vehicle group, with a decrease of cell number, sparse cell arrangements, and loss of integrity. In contrast, LUT treatment at 30 mg/kg, 60 mg/kg, and 90 mg/kg, but not 10 mg/kg, significantly improved neuronal survival after SAH (Figures 1(d) and 1(e)). There were no significant differences among 30 mg/kg, 60 mg/kg, and 90 mg/kg LUT treatment in ameliorating neurological deficits, brain edema, and neuronal degeneration after SAH (Figures 1(a)–1(e)). Based on the results of these tests, we found that 60 mg/kg was the optimum dosage. Therefore, we used this dose for the remaining experiments.

### 3.2. Effects of LUT on the Nrf2 Signaling Pathway and NLRP3 Inflammasome Activation after SAH

**Figure 2:**
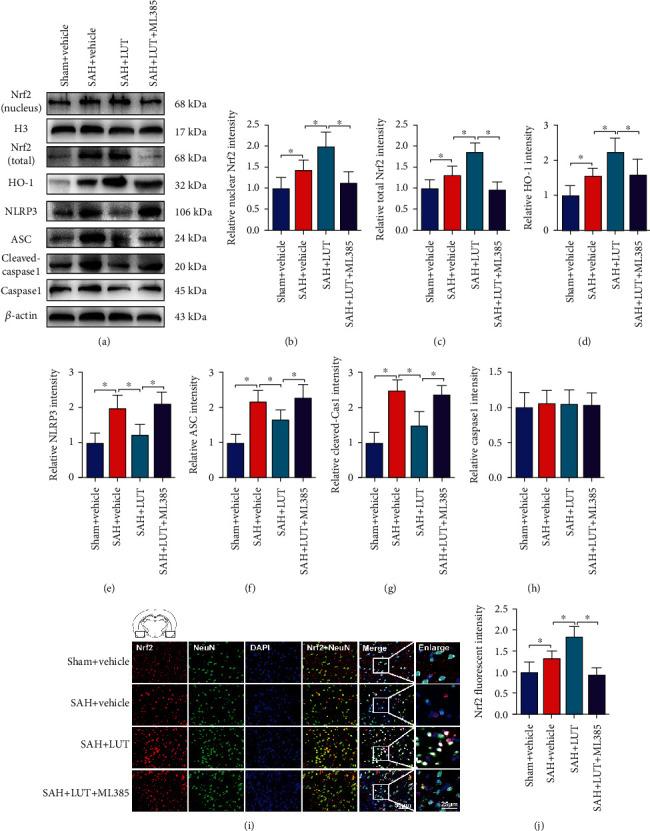
Effects of LUT treatment on Nrf2 and NLRP3 inflammasome signaling pathway after SAH. (a) Western blot assay for the expression of Nrf2, HO-1, NLRP3, ASC, cleaved caspase-1, and caspase-1 in the indicated groups. Quantitative analyses of (b) nucleus Nrf2, (c) total Nrf2, (d) HO-1, (e) NLRP3, (f) ASC, (g) cleaved caspase-1, and (h) caspase-1 expression in each group (*n* = 6 per group). (i, j) Representative photomicrographs and quantification of Nrf2 immunofluorescence staining (*n* = 6 per group). Magnification ×200, scale bar 50 *μ*m; (i) inset magnification ×400, scale bar 25 *μ*m. For immunofluorescence staining, the basal temporal lobe adjacent to the clotted blood was evaluated. Bars represent the mean ± SD. ^∗^*P* < 0.05.

LUT has been considered as a powerful Nrf2 activator in a variety of disorders. In addition, numerous studies have suggested that NLRP3 inflammasome activation is modulated by Nrf2 signaling [18, 19]. Thus, we used Western blot analysis to determine whether LUT induced Nrf2 activation and inhibited NLRP3 inflammasome signaling after SAH. ML385, a selective Nrf2 inhibitor, was further employed to inhibit Nrf2 signaling in this experiment. As shown in Figure 2, nuclear and total expression levels of Nrf2 protein and HO-1 protein were significantly increased after SAH, which could be further enhanced after LUT supplementation (Figures 2(a)–2(d)). In addition, SAH significantly induced the expression of NLRP3, ASC, and cleaved caspase-1, which was effectively inhibited by LUT administration (Figures 2(e)–2(h)). In contrast, ML385 pretreatment reversed LUT-induced Nrf2 expression and further increased protein levels of NLRP3, ASC, and cleaved caspase-1 (Figures 2(a)–2(h)). Double immunofluorescent staining confirmed the Western blot results, indicating that LUT enhanced nuclear translocation of Nrf2 after SAH, which was abrogated by LUT administration (Figures 2(i) and 2(j)). These data suggested that LUT could induce Nrf2 signaling and inhibit NLRP3 inflammasome activation after SAH.

### 3.3. Influence of LUT on Oxidative Damage at 24 h Post-SAH

**Figure 3:**
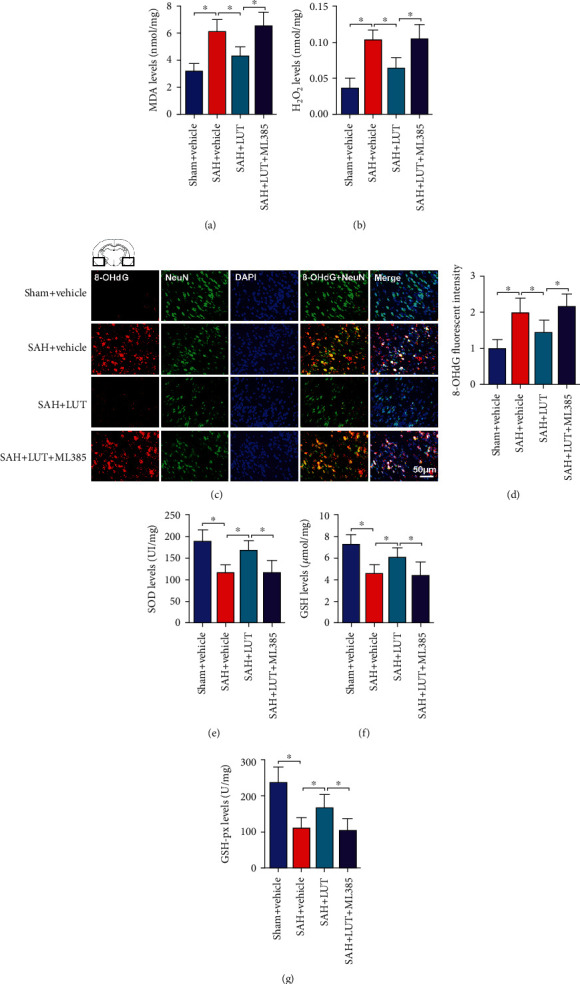
Effects of LUT on oxidative damage after SAH. Quantification of (a) MDA and (b) H_2_O_2_ levels in all groups (*n* = 6 per group). (c, d) Representative photomicrographs and quantification of 8-OHdG staining (*n* = 6 per group). (c) Magnification ×200, scale bar 50 *μ*m. For immunofluorescence staining, the basal temporal lobe adjacent to the clotted blood was evaluated. Quantification of (e) SOD, (f) GSH, and (g) GSH-Px in all groups (*n* = 6 per group). Bars represent the mean ± SD. ^∗^*P* < 0.05.

Nrf2 plays an important role in maintenance of redox homeostasis after SAH. We next evaluated whether LUT could ameliorate oxidative stress after SAH. As shown, SAH insults significantly induced oxidative damage, as evidenced by increases in lipid peroxidation, H_2_O_2_ generation, and 8-OHdG immunity (Figures 3(a)–3(d)). In addition, SAH markedly decreased the endogenous antioxidant systems including SOD, GSH, and GSH-Px activities as compared with those of the sham+vehicle group (Figures 3(e)–3(g)). In contrast, LUT administration significantly decreased oxidative insults and restored the impairment antioxidant systems after SAH (Figures 3(a)–3(g)). However, the antioxidant effects of LUT against SAH were abated by ML385 administration (Figures 3(a)–3(g)).

### 3.4. Influence of LUT on Neuroinflammation at 24 h Post-SAH

**Figure 4:**
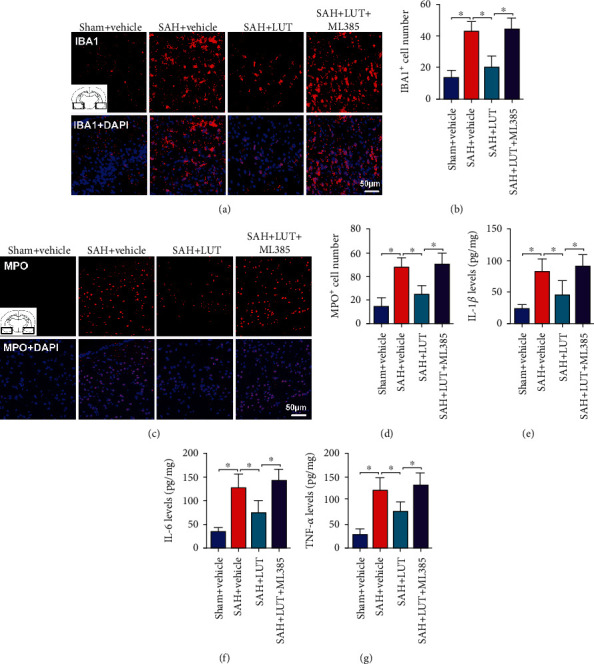
Effects of LUT on inflammatory response after SAH. Representative photomicrographs and quantification of (a, b) Iba-1 and (c, d) myeloperoxidase (MPO) staining (*n* = 6 per group). (a, c) Magnification ×200, scale bar 50 *μ*m. For immunofluorescence staining, the basal temporal lobe adjacent to the clotted blood was evaluated. Quantification of (e) IL-1*β*, (f) IL-6, and (g) TNF-*α* in all groups (*n* = 6 per group). Bars represent the mean ± SD. ^∗^*P* < 0.05.

NLRP3 inflammasome plays a critical role in initiating a series of immune-inflammatory reactions after SAH. We also evaluated whether LUT was able to mitigate neuroinflammation after SAH. The immunofluorescence staining showed that SAH insults significantly induced microglia activation and neutrophil infiltration when compared with those of the sham+vehicle group. LUT treatment significantly inhibited microglia activation and neutrophil infiltration after SAH, and that these effects were abolished by ML385 (Figures 4(a)–4(d)). In addition, SAH significantly induced the proinflammatory cytokine generation, which was suppressed by LUT treatment, while the decreased proinflammatory cytokine generation by LUT administration was reversed by Nrf2-specific inhibitor ML385 (Figures 4(e)–4(g)).

### 3.5. Effects of LUT on Neurologic Function and Neuronal Death after SAH

**Figure 5:**
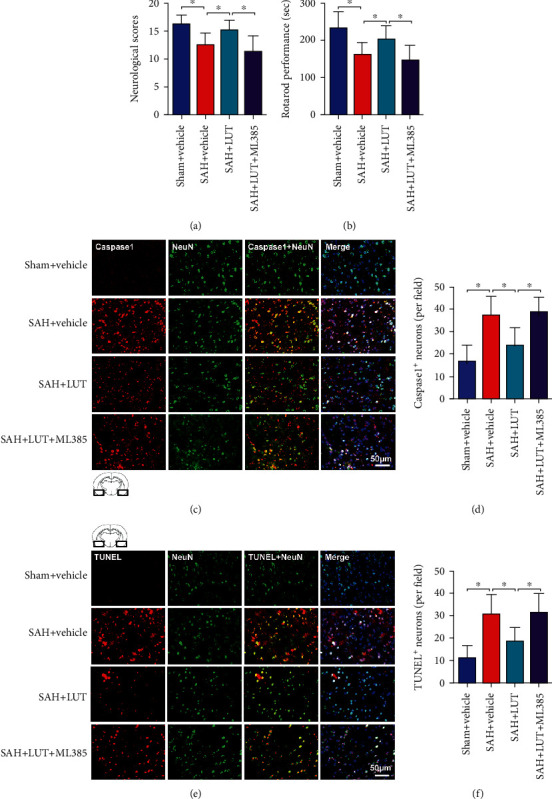
Effects of LUT on neurological function and neuronal death after SAH. Effects of LUT on (a) neurologic function and latency to fall in the (b) rotarod test after SAH (*n* = 12 per group). Representative photomicrographs and quantification of (c, d) caspase-1 and (e, f) TUNEL staining (*n* = 6 per group). (c, e) Magnification ×200, scale bar 50 *μ*m. For immunofluorescence staining, the basal temporal lobe adjacent to the clotted blood was evaluated. Bars represent the mean ± SD. ^∗^*P* < 0.05.

We next evaluated whether LUT could improve neurological function and ameliorate neuronal death. As shown, SAH caused significant neurologic impairment that was alleviated by LUT treatment (Figures 5(a) and 5(b)). In contrast, the beneficial effects of LUT on neurological function were reversed by ML385 pretreatment. Both neuronal apoptosis and pyroptosis contribute to the development of EBI after SAH. Cleaved caspase-1 is the canonical executor of pyroptosis. We further performed caspase-1 staining and TUNEL staining to examine neuronal pyroptosis and apoptosis, respectively. As shown, rats with SAH had greater numbers of caspase-1- and TUNEL-positive neurons when compared with sham-operated rats. LUT treatment significantly reduced the number of caspase-1- and TUNEL-positive neurons after SAH (Figures 5(c)–5(f)), while ML385 treatment reversed the LUT-induced decreases in neuronal apoptosis and pyroptosis after SAH (Figures 5(a)–5(f)).

### 3.6. Effects of LUT on Cell Viability, Oxidative Stress, and Inflammatory Response In Vitro

**Figure 6:**
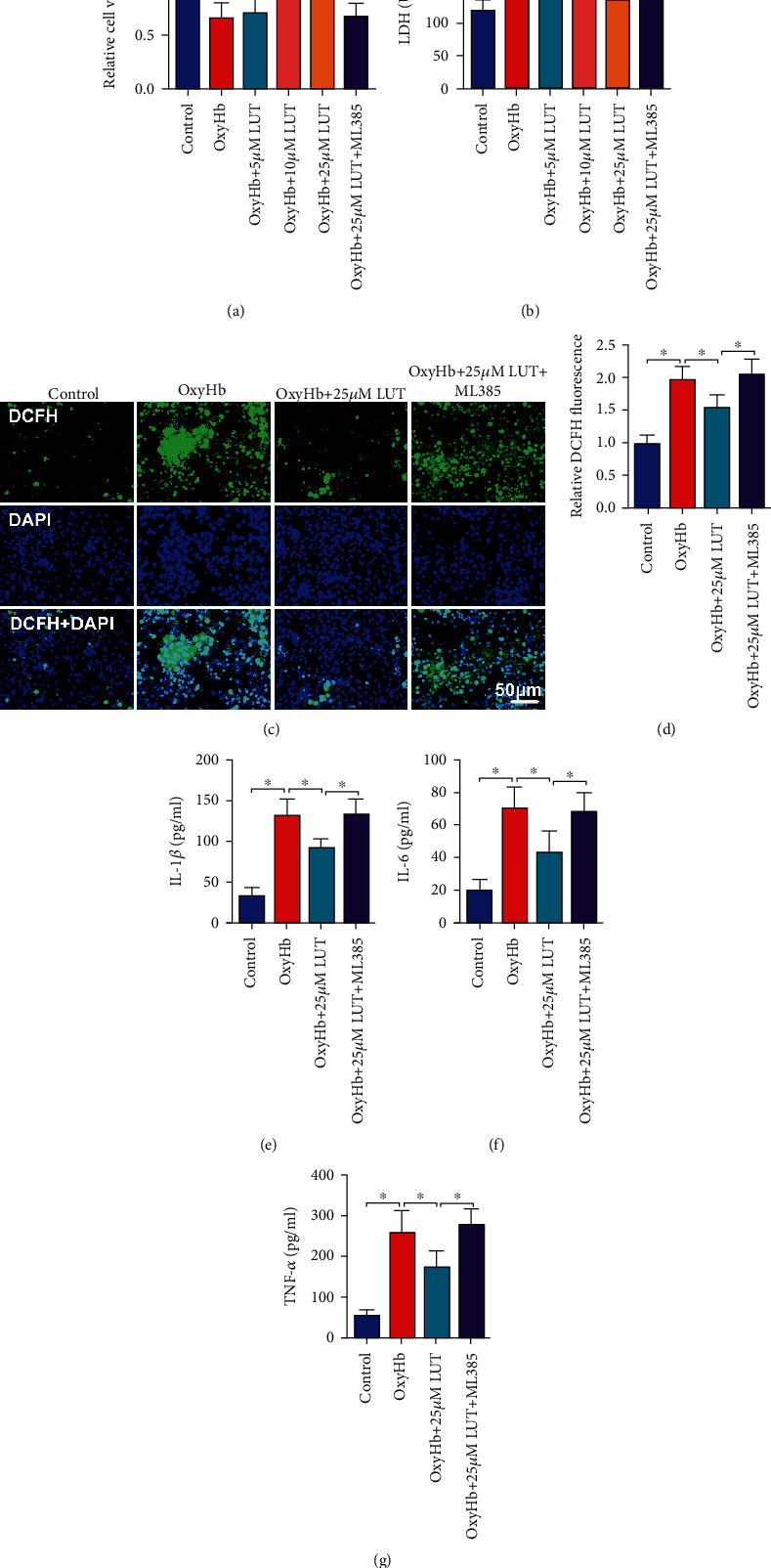
Effects of LUT treatment on oxidative stress, inflammatory response, and neuronal damage in vitro. Quantitative analysis of (a) cell viability and (b) LDH activity in the indicated groups (*n* = 6 per group). (c) Representative photomicrographs and (d) quantification of DCFH immunofluorescence (*n* = 6 per group). (c) Magnification ×200, scale bar 50 *μ*m. Quantification of (e) IL-1*β*, (f) IL-6, and (g) TNF-*α* in culture medium (*n* = 6 per group). Bars represent the mean ± SD. ^∗^*P* < 0.05.

We further confirmed the potential beneficial effects of LUT in vitro. As shown, our data indicated that LUT significantly improved neuronal viability, reduced oxidative damage, and decreased expression of proinflammatory cytokines including IL-1*β*, IL-6, and TNF-*α* after OxyHb stimulation (Figures 6(a)–6(g)). However, all these changes were abrogated by ML385 treatment (Figures 6(a)–6(g)).

### 3.7. Effects of LUT on Nrf2 and NLRP3 Inflammasome Signaling In Vitro

**Figure 7:**
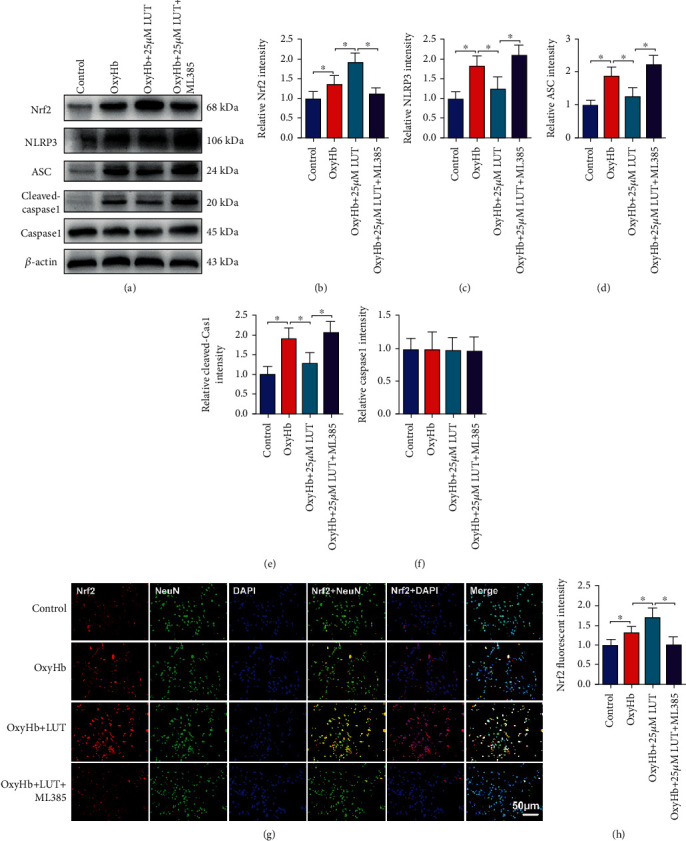
Effects of LUT treatment on Nrf2 and NLRP3 inflammasome activation in vitro. (a) Representative Western blots and quantification of expression of (b) Nrf2, (c) NLRP3, (d) ASC, (e) cleaved caspase-1, and (f) caspase-1 (*n* = 6 per group). (g) Representative photomicrographs of Nrf2 immunofluorescence staining. (g) Magnification ×200, scale bar 50 *μ*m. (h) Quantification of Nrf2 immunofluorescence staining in all experimental groups (*n* = 6 per group). Bars represent the mean ± SD. ^∗^*P* < 0.05.

We next evaluated the effects of LUT on Nrf2 and NLRP3 inflammasome signaling in vitro. Western blot analysis showed that LUT treatment significantly increased the expression of Nrf2 and reduced expression of NLRP3, ASC, and cleaved caspase-1 in primary microglia and neurons exposed to OxyHb. These changes were abrogated by Nrf2-selective inhibitor ML385 (Figures 7(a)–7(e)). Double immunofluorescence staining further showed that LUT enhanced the expression of Nrf2 in primary cortical neurons, which was abated by ML385 treatment (Figures 7(f) and 7(g)).

### 3.8. Effects of LUT on Neuronal Degeneration In Vitro

**Figure 8:**
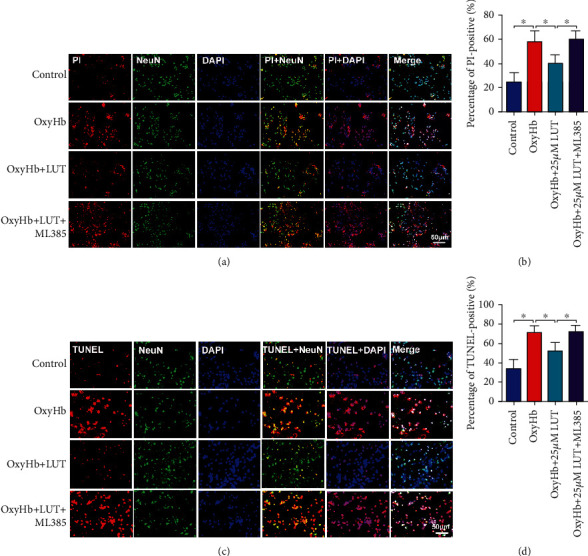
Effects of LUT treatment on PI staining and TUNEL staining in vitro. (a) Representative photomicrographs of PI immunofluorescence staining. (b) Quantification of PI staining in primary cortical neurons (*n* = 6 per group). (c) Representative photomicrographs of TUNEL immunofluorescence staining. (d) Quantification of TUNEL staining in primary cortical neurons (*n* = 6 per group). (a, c) Magnification ×200, scale bar 50 *μ*m. Bars represent the mean ± SD. ^∗^*P* < 0.05.

It has demonstrated that both neuronal apoptosis and pyroptosis contribute to the development of EBI after SAH. PI cannot pass intact plasma membrane and can only be present in DNA of cells where the plasma membrane has been compromised. During pyroptosis, pores can be formed in the cell membrane and can be detected by PI staining [26]. TUNEL staining can detect the DNA breaks when DNA fragmentation occurs in the last phase of apoptosis. Therefore, in this experiment, we used PI and TUNEL staining to examine neuronal pyroptosis and apoptosis, respectively. As shown, OxyHb stimulation significantly increased the number of PI- and TUNEL-positive neurons, which were decreased by LUT treatment (Figures 8(a)–8(d)). In contrast, the improvement by LUT was reversed by ML385 administration (Figures 8(a)–8(d)). These suggested that LUT could reduce neuronal pyroptosis and apoptosis in vitro.

## 4. Discussion

Neuroinflammation and oxidative damage are two major etiological factors resulting in the secondary brain injury after SAH [27–29]. After the initial hemorrhage, blood components enter the subarachnoid space and activate innate and adaptive immune cascade responses. Microglia activation and neutrophils recruit to the damaged tissue and release a variety of inflammatory factors that exacerbate neurons [30]. Excessive ROS can elicit neuronal damage by promoting lipid peroxidation and DNA damage. Moreover, the robust inflammatory response produces additional excess ROS, further aggravating the redox imbalance and thereby inducing neuronal cell death and neurological deficits after SAH [31–33]. Accordingly, pharmacologically reducing neuroinflammation and oxidative damage might provide a means to ameliorate SAH.

In recent years, mounting evidence has suggested that LUT is a promising neuroprotective agent in a variety of neurological disorders [6]. It has been demonstrated that LUT has a wide range of pharmacological properties including antioxidant free-radical scavenging and anti-inflammatory effects [20, 34, 35]. For example, Kou et al. reported that LUT alleviated cognitive impairment in Alzheimer's disease mouse model via inhibiting endoplasmic reticulum stress in astrocytes and subsequent neuroinflammation [36]. Tan et al. demonstrated that LUT provided autophagy and antioxidative effects in both in vivo and in vitro models of intracerebral hemorrhage [7]. However, to our knowledge, no study has yet investigated the potential effects of LUT in experimental SAH and the underlying molecular mechanisms.

Nrf2 as a critical translate factor in maintenance of redox homeostasis is widely studied in recent years. After stimulation by oxidative stress, Nrf2 translocates into the nucleus and binds to the ARE, subsequently inducing antioxidant enzyme expression [37, 38]. Loads of evidence have indicated that Nrf2 signaling activation could significantly ameliorate SAH-induced oxidative damage and brain injury [39, 40]. Intriguingly, LUT has been considered as a powerful Nrf2 activator in a variety of disorders [7, 8]. Xu et al. reported that LUT could provide neuroprotective effects in TBI models both in vivo and in vitro through the activation of Nrf2-ARE pathway [8]. Xiao et al. indicated that LUT attenuated cardiac ischemia/reperfusion injury in diabetic rats by activation of Nrf2 signaling [41]. Thus, it is reasonable to predict that LUT can activate Nrf2 signaling and confer cerebroprotective effects in SAH. It has been clarified that Nrf2 can transcriptionally induce numerous antioxidative genes including HO-1, GSH-Px, and SOD. HO-1 plays a prominent role in maintenance of cellular homeostasis by degrading heme [42]. SOD and GSH-Px are antioxidant enzymes which play fundamental and indispensable role in against free radical attack [43]. Consistent with these previous studies, we found that LUT also markedly enhanced the translocation of Nrf2 into the nucleus and Nrf2-involved antioxidative enzyme expression. Meanwhile, LUT significantly reduced the brain MDA and 8-OHdG contents after SAH insults. MDA and 8-OHdG are two key biomarkers for lipid peroxidation and DNA oxidative damage caused by excessive ROS, respectively. These results indicated that LUT could protect against SAH injury through scavenging ROS and enhancing the endogenous antioxidative system by the modulation of Nrf2 signaling.

Another interesting finding in the current study was that LUT ameliorated neuroinflammation and inhibited NLRP3 inflammasome signaling activation. NLRP3 inflammasome is a cytoplasmic multiprotein complex of the innate immune system that can initiate a series of immune-inflammatory reactions [44]. It has been proved that NLRP3 inflammasome-mediated neuroinflammation plays a prominent role in the secondary brain injury after SAH [11, 12]. Once activated, NLRP3 inflammasome cleaves procaspase-1 resulting in pro-IL-1*β* and pro-IL-18 activation. The inflammatory cytokines can further activate immune-related cells, such as neutrophils, to generate the corresponding immune effects. In addition to amplify inflammation, cleaved caspase-1 is the canonical executor of pyroptosis to further exacerbate neuronal cell death after SAH [10]. According to recent studies, LUT is able to inhibit NLRP3 inflammasome activation in different research fields [20, 21]. In our current study, we also observed that LUT significantly suppressed NLRP3 inflammasome activation after SAH and the subsequent inflammatory response, including microglia activation, neutrophil infiltration, and inflammatory cytokine release. In addition, the SAH-induced neuronal apoptosis and pyroptosis were markedly reduced after LUT administration. However, the underlying mechanism that mediates the inhibition of NLRP3 inflammasome by LUT in SAH needs to be elucidated.

It is known that ROS production is one of the major signals that trigger the NLRP3 inflammasome activation [13]. Both ROS overproduction and neuroinflammation are considered crucial elements of EBI after SAH, and each of them promotes and amplifies the other one [23]. Under normal conditions, the basal level of the NLRP3 inflammasome is low in immune cells. Upon stimulation by ROS, the NLRP3 inflammasome is assembled and activated to amplify the innate immune response after SAH [23]. Previous studies have proved that decreasing ROS overproduction could prevent NLRP3 inflammasome activation and mitigate the elevated levels of proinflammatory cytokine release in different neurological disorders, thereby ameliorating neuroinflammation [13, 23, 45]. As discussed above, we hypothesized that LUT might affect ROS production by modulating Nrf2 activation, thereby preventing NLRP3 inflammasome signaling after SAH. The relationship between Nrf2 signaling and NLRP3 inflammasome in other research fields has been well discussed in a variety of studies in recent years [46, 47]. Mounting evidence has demonstrated that Nrf2 activation could inhibit NLRP3 inflammasome by inducing numerous antioxidative genes including HO-1, thereby scavenging ROS production [48, 49]. HO-1, one of the cytoprotective enzymes induced by the Nrf2-ARE pathway, has been widely regarded as a protective mechanism against oxidative stress and ROS [50, 51]. It has been proved that increased HO-1 expression could remove ROS and maintenance of the internal cellular environment. For example, Seiwert et al. reported that HO-1 protects human colonocytes against ROS formation, oxidative DNA damage, and cytotoxicity induced by heme iron [52]. Interestingly, we also observed that LUT treatment significantly increased HO-1 expression after SAH, suggesting that HO-1 might be involved in the inhibitory effects of LUT on NLRP3 inflammasome activation. To further address this hypothesis, we employed a novel and selective Nrf2 inhibitor ML385 in our study [53]. As expected, we observed that ML385 pretreatment significantly abated LUT-induced Nrf2 signaling-mediated HO-1 expression, ROS suppression, and NLRP3 inflammasome inhibition. Additionally, ML385 treatment abrogated the neuroprotective effects of LUT against SAH-induced oxidative damage, neuroinflammation, neuronal apoptosis, and pyroptosis. These findings supported the notion that LUT inhibited NLRP3 inflammasome activation might involve Nrf2 signaling pathway. Based on these outcomes, we further evaluated the effects of LUT in an in vitro SAH model. In agreement with the results in vivo, LUT inhibited neuroinflammation, oxidative damage, and neuronal apoptosis and pyroptosis in vitro, which were associated with NLRP3 inflammasome inhibition and Nrf2 activation and were reversed by ML385. Taken together, our data indicated that the cerebroprotective effects of LUT might be attributed to its ability to induce Nrf2 activation and thereby inhibiting the NLRP3 inflammasome signaling.

However, there are several limitations in our study. Firstly, in the dose-response experiments, we cannot conclude that 60 mg/kg LUT is the optimum dose to provide maximal effect. Although there were no statistical differences between 30 mg/kg and 60 mg/kg LUT in ameliorating neurological deficits and neuronal degeneration, we can still see that 60 mg/kg LUT had a better act on neurological outcomes and neuronal survival after SAH. A larger sample size might decipher these discrepancies. Additionally, the toxicity studies should be further conducted to verify the optimal dose of LUT in SAH. Secondly, how LUT regulates Nrf2 activation remains unclear. Some possible mechanisms might be involved in this action, such as sirtuin 1 (SIRT1), adenosine monophosphate-activated protein kinase (AMPK), and PI3K [14–16, 54]. Yang et al. reported that dietary LUT could attenuate HgCl_2_-induced liver dysfunction by regulating SIRT1/Nrf2/TNF-*α* signaling pathway. Another two previous studies have indicated that LUT could significantly increase levels of PI3K and phosphorylated AMPK to activate Nrf2 pathway in different research areas [14, 55]. However, whether these molecular targets are attributable to the activation of Nrf2 signaling by LUT after SAH is needed to be clarified. Lastly, it should be noted that in addition to HO-1, Nrf2 can transcriptionally induce a variety of antioxidative genes including NAD(P) H dehydrogenase quinone 1 (NQO-1). It has been demonstrated that NQO-1 plays a critical role in monitoring cellular redox state and protects against oxidative stress induced by a variety of metabolic situations [56, 57]. Whether NQO-1 is involved in the beneficial effects of LUT against SAH remains obscure. Given that the present research is a pilot study, further experiments are still needed to validate the exact role of LUT following SAH.

## 5. Conclusion

In conclusion, we provide the first evidence that LUT exerts cerebroprotective effects against SAH by inhibiting NLRP3 inflammasome activation, which may be largely dependent on upregulation of the Nrf2 signaling pathway. LUT may serve as a promising candidate for SAH treatment.

## Data Availability

All the data supporting the results were shown in the paper and can be applicable from the corresponding author upon reasonable request. Some data may not be made available because of privacy or ethical restrictions.

## Abbreviations

8-OHdG: 8-Hydroxydeoxyguanosine
AMPK: Adenosine monophosphate-activated protein kinase
ARE: Antioxidant response element
ASC: Adaptor apoptosis-related speck-like protein
DCFH-DA: 2,7-Dichlorodihydrofluorescein diacetate
DMSO: Dimethylsulfoxide
GSH: Glutathione
GSH-Px: Glutathione peroxidase
H_2_O_2_: Hydrogen peroxide
LDH: Lactate dehydrogenase
LUT: Luteolin
MDA: Malondialdehyde
NLRP3: Nod-like receptor pyrin domain-containing 3
NQO-1: NAD(P)H dehydrogenase quinone 1
Nrf2: Nuclear factor-erythroid 2-related factor 2
OxyHb: Oxyhemoglobin
PI: Propidium iodide
ROS: Reactive oxygen species
SAH: Subarachnoid hemorrhage
SIRT1: Sirtuin 1
SOD: Superoxide dismutase
TBI: Traumatic brain injury
TNF-*α*: Tumor necrosis factor-*α*
TUNEL: Terminal deoxynucleotidyl transferase-mediated dUTP nick-end labeling.
